# Exploring breast and prostate cancer RNA-seq derived radiosensitivity with the Genomic Adjusted Radiation Dose (GARD) model

**DOI:** 10.1016/j.ctro.2022.08.002

**Published:** 2022-08-09

**Authors:** Ben Nolan, Brian O’Sullivan, Aaron Golden

**Affiliations:** a Discipline of Bioinformatics, School of Mathematical and Statistical Sciences, National University of Ireland Galway, University Road, Galway H91 TK33, Ireland; b School of Natural Sciences, National University of Ireland Galway, University Road, Galway H91 TK33, Ireland

**Keywords:** Radiation therapy, Genomics, Personalized medicine, Prostate cancer, Breast cancer, GARD

## Abstract

The use of a 10 gene transcriptional signature as part of the GARD model has been shown to be predictive of radiotherapy benefit for a range of cancers, with the potential to determine an optimal overall dose per patient. We used publicly available RNA-seq transcriptomics data from a luminal B breast cancer patient and from 14 prostate cancer patients to explore the radiosensitivity indices (RSI) and so GARD estimates of both tumour and proximal normal biopsies from each individual. Clear differences of clinical relevance in derived radiobiological properties between tumour and proximal normal tissues were evident for the breast cancer patient, whilst such differences across the prostate cancer cohort were more equivocal. Using the prostate cancer cohort’s median tumour predicted GARD value as a threshold for high therapeutic effect for radiotherapy, we found evidence that a higher overall prescribed dose than the widely used 72 Gy/36fx could benefit half of these patients. This exploratory study demonstrates the potential combining the GARD model with sequencing based transcriptomics could have in informing personalised radiotherapeutic practise for both breast and prostate cancer patients.

## Highlights

• RNA-seq data taken from patient tumor and normal samples are used to derive GARD (Genome Adjusted Radiation Dose) radiosensitivity indices for a luminal B breast cancer patient (n = 1), and a cohort of prostate cancer patients (n = 14)
• Clear differences in radiosensitivity were evident between tumor and normal tissues for the luminal B breast cancer patient
• A treatment of 50 Gy/25fx would yield a GARD value passing the threshold for an optimal therapeutic index for this breast cancer patient
• The majority of prostate cancer patients show tumour radiosensitivity differences and so potential suitability for hypofractionation
• A treatment of 72 Gy/36fx results in variable GARD values for the prostate cancer patients, highlighting the benefit of higher optimal dosages in some cases

## 1 Introduction

Despite the widespread use and acknowledged efficacy of radiotherapy in treating patients with a range of cancers, there is a growing appreciation that the standardised treatment regimen prescribed for specific tumours may be sub-optimal. The one-size-fits-all approach is unlikely to have an equal therapeutic effect for all patients due to patient genotype and tumour heterogeneity. The growth in the use of genomics assays over the past decade has provided a means to formally assess radiosensitivity at the individual level. Work studying clonogen survival curves from a subset of the NCI60 panel together with associated transcriptional activity culminated in the derivation of a 10-gene panel based radiosensitivity index (RSI), measured in units of SF2, which was validated in several clinical studies [1], demonstrating enhanced radiotherapeutic efficacy based on a given tumour’s transcriptomic derived RSI.

Subsequent work incorporating the predicted transcriptional RSI into the Linear Quadratic formalism yielded the development of the Genome Adjusted Radiation Dose (GARD) model [2], which permits estimation of patient specific radiobiological properties directly from the expression profile of the tumour under scrutiny. Several subsequent studies have further validated the efficacy of the GARD model in quantifying the efficacy of radiotherapy for certain patients over others for several cancer types, and in using the distribution of GARD estimates among a given cohort to identify those patients for whom a lesser or greater overall radiation dose would be appropriate to optimise tumour control [3], [4]. In the most recent study, a pooled pan-cancer analysis in 11 separate clinical cohorts of 1,615 unique patients with 7 different cancer types definitely demonstrated the GARD derived dose, not overall physical RT doses, was predictive of RT benefit as regards recurrence and overall survival [5].

Quantifying normal tissue complications is a critical component of any radiation treatment plan, and by convention this is determined from agreed tissue-specific radiobiological parameters that are considered universally applicable to all patients. However, the personalised and fundamentally genomic nature of radiotoxicity has been known for some time [6], [7], [8]. The conventional GARD formalism lacks a means of quantifying radiosensitivity for proximal normal tissues to the tumour being targeted, as the RSI is typically estimated from expression microarray analyses which are based around a differential estimate of expression between pair-matched tumour and normal tissue samples. Indeed, questions have been raised about the potential presence of normal tissue within the tumour samples compromising the GARD assay itself [9]. We reasoned that by directly sequencing transcripts from both tumour and normal tissue samples using RNA-seq, we could implement the GARD model for both separately, and use this information to assess the suitability of radiation dose escalation for a given patient, in particular for those cases where limiting proximal normal tissue radiotoxicity is a priority.

In this short communication, we articulate an exploratory study involving publicly available RNA sequence data for one breast cancer patient (T  = 10, N  = 3) where we describe our novel methodology to generate RSI estimates directly from such transcriptomic data, and how we use these to characterise the likely radiotherapeutic response - both tumour and proximal normal tissue - of this patient to a standard fractionation treatment regimen of 50 Gy/25fx. We additionally apply the same approach to a publicly available RNA sequence data archive for 14 prostate cancer patients (T = 1, N = 1 for each patient). As the radiotoxicity of proximal normal prostate tissue is not of current clinical relevance, our interest here is to determine if dose escalation would be appropriate for all/any of these patients as derived from each tumour’s RSI. As no prior studies involving prostate cancer have been reported using the GARD methodology, this component of our exploratory study is particularly novel.

## 2 Materials and Methods

### 2.1 Sequence Data

10 tumour and 3 adjacent normal samples were biopsied from a single Korean woman diagnosed with invasive ductal luminal B carcinoma, and the extracted RNA sequenced using ∼100 bp paired-end reads on an Illumina HiSeq 2500 platform [10], [11]. Patient metadata can be found in Supplementary Table S1. For the prostate cancer cohort, data was retrieved from a previously published study examining and building a transcriptomic landscape for prostate cancer from 14 patients, in this case the biopsies obtained following radical prostatectomy [12]. Data for each patient contains 1 tumour and 1 adjacent normal sample. RNA was sequenced with 90 bp paired-end reads on the Illumina HiSeq 2000 platform. Patient metadata can be found in Supplementary Tables S2 & S3.

### 2.2 Analysis

All sequence data were assessed for quality control using FastQC/MultiQC [13], [14] and RseQC package [15], and aligned to Ensembl Version 104 of the human genome using HISAT2 [16]. Differential expression was determined using kallisto [17] and DESeq2 [18]. PCA analysis of gene expression variation within both cohorts following regularized-logarithm transformation was used to identify poor quality samples. The resulting PCA and Transcription Integrity Number (TIN) plots can be found in Supplementary FiguresS1 & S2. All processing was implemented using Nextflow scripting [19] curated within a Docker/Singularity container permitting ease of reproducibility [20]. Gene level transcript count data was normalized to counts per million using edgeR [21], followed by trimmed means of M (TMM) values, a normalization method which allows comparison across multiple samples [22].

The radiosensitivity index (RSI) was calculated directly from the expression values determined for the 10-gene panel of [1] using the following formula:

(1) RSI=-0.0098009*AR+0.0128283*cJun+0.0254552*STAT1-0.0017589*PKC-0.0038171*RelA+0.1070213*cABL-0.0002509*SUMO1-0.0092431*CDK1-0.0204469*HDAC1-0.0441683*IRF1

The Genomic-Adjusted Radiation Dose (GARD) can be calculated as defined [23] using the α value determined by the radiosensitivity index, and β as a fixed 0.05, using the following formula:

(2) GARD=nd(α+βd)

where the α parameter determined as follows:

(3) $$\alpha =\frac{\ln(\mathrm{RSI})+\beta {\mathrm{nd}}^{2}}{-\mathrm{nd}}=(-0.5\ln(\mathrm{RSI}))-0.1$$

where *n* are the number of fractions of dose *d*. Conversely, for a given GARD value, one can estimate the optimal radiation dose, and so using a GARD threshold value, derive the optimum dose per patient.

All statistical evaluation was performed using R [24], [25]. RSI and GARD variance for both breast cancer and prostate cancer datasets were calculated using Welch’s T-test.

### 2.3 Results

**Fig. 1:**
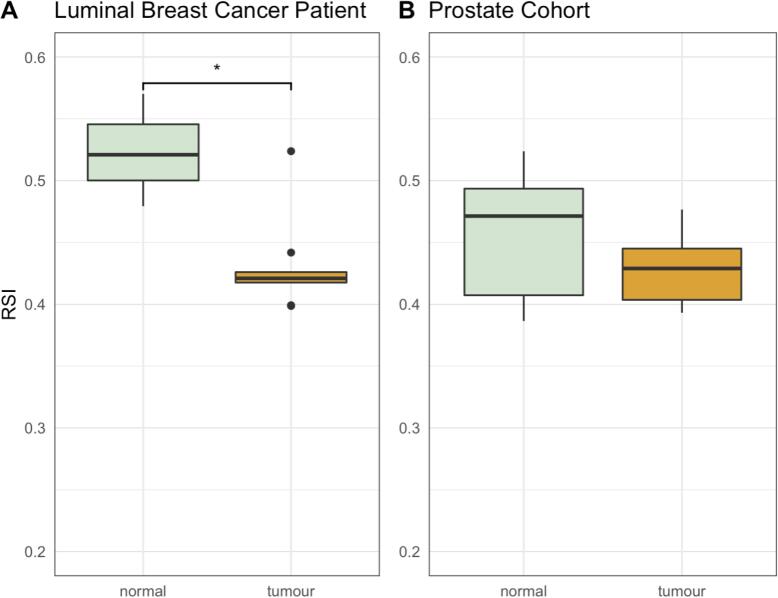
**A**: Luminal B Breast Cancer Patient: Boxplot showcasing RSI for 9 tumour and 3 adjacent normal tissue samples. **B**: Prostate Cancer Patient: Boxplot showcasing RSI for tumour and adjacent normal tissue in 14 normal-tumour matched samples.

After quality control measures, the breast cancer dataset was reduced to T = 9, N = 3, following the removal of one outlier tumour sample from this study (Supplementary Figure S1). RSI was measured and resulted in values of 0.43 ± 0.038 and 0.52 ± 0.045 for tumour and normal tissue samples respectively for the luminal B breast cancer patient (P = 0.049)(Fig. 1A), with values of 0.43 ± 0.024 and 0.46 ± 0.049 for tumour and normal tissue samples respectively for the prostate cancer patient (P = 0.073)(Fig. 1B). The same data represented as both scatter and bar plots may be found in Supplementary Figures S3 & S4.

We hypothesised a standard-of-care dose RT of 50 Gy in 25 fractions to derive GARD values for the breast cancer case, yielding a GARD value of 21.18 ± 2.04 and 16.24 ± 2.17 for the tumour and normal tissue samples respectively (P = 0.035). This matches the threshold derived by [5] as demonstrating a positive therapeutic index, indicating such a fractionation regimen would have been appropriate for this patient. We similarly applied a hypothetical standard-of-care RT of 72 Gy in 36 fractions for each of the prostate samples, however there was no statistical difference between the derived cancer/normal GARD values (30.66±2.05/28.54±3.92, P = 0.088).

**Fig. 2:**
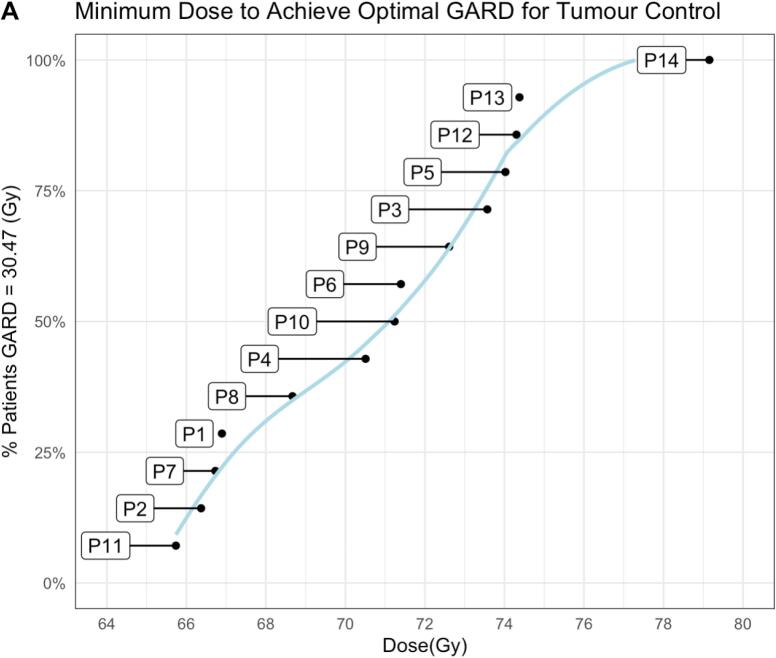
**A:** Empirical cumulative distribution plot for the minimum total radiotherapy dose for tumour control per patient in the prostate dataset, using median GARD across the prostate dataset as the threshold for a high GARD score. The labels indicate each patient’s GARD value in the plot.

We used the median predicted GARD value for the tumour samples to define a threshold for GARD ‘high’ predictive of a high therapeutic effect, as previously proposed by [23], and derived an empirical distribution function plot (Fig. 2A) for each patient. A LOESS model fit through the optimal patient dosages yields a sigmoidal curve, as predicted from tumour control probability (TCP) models and previously demonstrated in [26], [27], where the sigmoidal distribution was generated from a TCP model using Gaussian distributed α values at 0.35 with 0.08 standard deviation, suggesting a similar relationship between α values and optimal patient dosage using the GARD model. Fig. 2A highlights the benefit higher overall dosages could have for several patients, in some cases greater than the standard prescribed (72 Gy/36fx). 35% of patients would achieve a high TCP at 70 Gy dosage in 2 Gy fractions, with 92% of patients obtaining high TCP at 75 Gy.

## 3 Discussion

This study aimed to create a RNA-seq based methodology for RSI calculation and utilize the GARD model in order to assess the efficacy of planned standard of care radiotherapy on an individualized basis, taking into account adjacent normal tissue radiosensitivity as required. The latter is of particular importance for those cancers localised within existing organs for which there is a need to limit radiotoxicity, such as breast cancers. The use of RNA-seq data distinguishes it from the conventional GARD estimation which is based on a gene expression microarray methodology. Recent work by Dai et al. [28] attempted to determine RSI directly from RNA-seq data, however that approach could not correct for cross-sample count normalization [29]. By applying a weighted trimmed means of M (TMM) values with log2 transformation, we have been able to correct for both inter-sample gene count comparison and cross sample normalization [30].

We used this methodology to explore the radiosensitivity heterogeneity across tumour and adjacent normal tissue in two datasets, in a single patient with luminal B breast cancer and in 14 patients with prostate cancer. In both cases, the gene panel derived RSI values showed greater consistency for the tumour against the normal samples for both cancer types, in addition to an overall trend for greater RSI associated with normal tissues. Whilst more forensic analysis would be necessary to confirm normal tissue ‘contamination’ in the tumour samples, its clear that the differences in RSI are consistent with differing transcriptional signatures, consistent with our gene expression analysis (data not shown), resolving to some extent prior concerns of normal tissue contamination compromising the GARD model [9].

For the luminal B breast cancer dataset, both RSI and GARD estimates were significantly different between the tumour and normal tissue biopsies from the same patient, with the GARD value at the 21 threshold previously reported for enhanced radiotherapeutic efficacy [5]. The significant difference in GARD derived optimal dose between both breast tissue types indicates the potential for enhanced tumour control probability by increasing the overall biological effective dose without enhancing normal toxicity. Optimal radiotherapeutic regimens aim to maximise the therapeutic index between tumour control and normal tissue complication probabilities. This preliminary study demonstrates the potential of using GARD to characterise the predicted radiotherapeutic outcome of both the tumour and proximal normal tissue for a given patient, and in so doing, provide valuable additional information towards personalising treatment.

Limiting radiotoxicity to proximal normal prostate tissue is not considered in clinical practise, and so the variations in tumour-normal RSI determined for each patient in the prostate cancer cohort are not in any way actionable, other than pointing to clearly differing radiobiological conditions between both tissue types. The majority of patients in the prostate cancer cohort show tumour RSI values in excess of 0.4, which has been previously proposed as being a threshold indicating suitability for hypofractionation[31], [32] (Supplementary FigureS4). Many of these same patients were also identified as likely requiring a higher prescribed dose to achieve optimal GARD for tumour control in our study (Fig. 2). Of note is the fact that there is no evident correlation between the RSI estimates and other clinical variables such as PSA, stage, or Gleason Score (Table S1). Whilst we caution drawing conclusions on such a small sample, taken together these data suggest that such RSI profiling may have value in radiation treatment planning for treating localised disease in this first application of the GARD model to prostate cancer.

## Ethical approval and consent to participate

This study involved the use of publicly available RNA-seq data for which prior ethical approval and consent would have been required prior to submission on the BioProject repository.

## Consent for publication

Not applicable.

## Availability of data and materials

The breast cancer data is available at BioProject: PRJNA432903; GEO:GSE110114. The prostate cancer data is available at BioProject: PRJEB2449. All code used to process data is available on github: https://github.com/BenNolann/rsi_analysis. A docker container with all relevant software: https://hub.docker.com/repository/docker/bennolan/rnaseq

## Authors’ contributions

AG conceived of the study, detailed the proposed work, coordinated the study, and lead the drafting of the manuscript. BN and BOS worked equally on processing and analysing the data, and contributed to the drafting of the manuscript.

## Declaration of Competing Interest

The authors declare that they have no known competing financial interests or personal relationships that could have appeared to influence the work reported in this paper.
