# Supplementary material for: Exploring breast and prostate cancer RNA-seq derived radiosensitivity with the Genomic Adjusted Radiation Dose (GARD) model
Source: Clin Transl Radiat Oncol. 2022 Aug 9;36:127–31. doi: 10.1016/j.ctro.2022.08.002 (PMC9396042; doi:10.1016/j.ctro.2022.08.002)
Supplement: Supplementary data 1 [file mmc1.pdf]

9. Appendix A. Supplementary data

Table S1: Luminal Breast Cancer Patient Sample Data

Accession	Condition	Replicate	Reference ID	Notes
SRR6671104	tumour	1	T0	
SRR6671105	tumour	2	T1	
SRR6671106	tumour	3	T2	
SRR6671107	tumour	4	T3	Failed QC
SRR6671108	tumour	5	T4	
SRR6671109	tumour	6	T5	
SRR6671110	tumour	7	T6	
SRR6671111	tumour	8	T7	
SRR6671112	tumour	9	T8	
SRR6671113	tumour	10	T9	
SRR6671114	normal	1	N1	
SRR6671115	normal	2	N2	
SRR6671116	normal	3	N3	

Table S2: Prostate Patient Clinical Data

Patient ID	Age	Preoperative PSA	Stage	Gleason Score	Metastasis
1	74	9.85	T2cN0M0	3+4	0
2	73	1.36	T1cN0M0	3+3	0
3	71	9.62	T2aN0M0	2+2	0
4	54	7.44	T2cN0M0	3+3	0
5	62	7.76	T4N0M0	3+4	Bladder
6	69	4.04	T1cN0M0	3+4	0
7	52	30.33	T3bN0M0	3+4	0
8	66	10.4	T3aN0M0	3+4	0
9	56	9.78	T2cN0M0	3+4	0
10	75	10.93	T2cN0M0	3+4	0
11	57	6.99	T2cN0M0	3+4	0
12	80	22.38	T1cN0M0	4+3	0
13	75	12.69	T4N0M0	5+3	Bladder
14	73	12.8	T2bN0M0	3+2	0

Table S3: Prostate Cancer Patient Sample Data

Accession	Condition	Replicate	Title	Patient ID
ERR299295	tumour	1	11T	11
ERR299296	normal	1	12N	12
ERR299297	tumour	1	4T	4
ERR299298	tumour	1	5T	5
ERR299299	normal	1	6N	6
ERR031017	normal	1	10N	10
ERR031018	tumour	1	10T	10
ERR031019	normal	1	11N	11
ERR031022	tumour	1	12T	12
ERR031023	normal	1	13N	13
ERR031024	tumour	1	13T	13
ERR031025	normal	1	14N	14
ERR031026	tumour	1	14T	14
ERR031027	normal	1	1N	1
ERR031028	tumour	1	1T	1
ERR031029	normal	1	2N	2
ERR031030	tumour	1	2T	2
ERR031031	normal	1	3N	3
ERR031032	tumour	1	3T	3
ERR031033	normal	1	4N	4
ERR031035	normal	1	5N	5
ERR031038	tumour	1	6T	6
ERR031039	normal	1	7N	7
ERR031040	tumour	1	7T	7
ERR031041	normal	1	8N	8
ERR031042	tumour	1	8T	8
ERR031043	normal	1	9N	9
ERR031044	tumour	1	9T	9

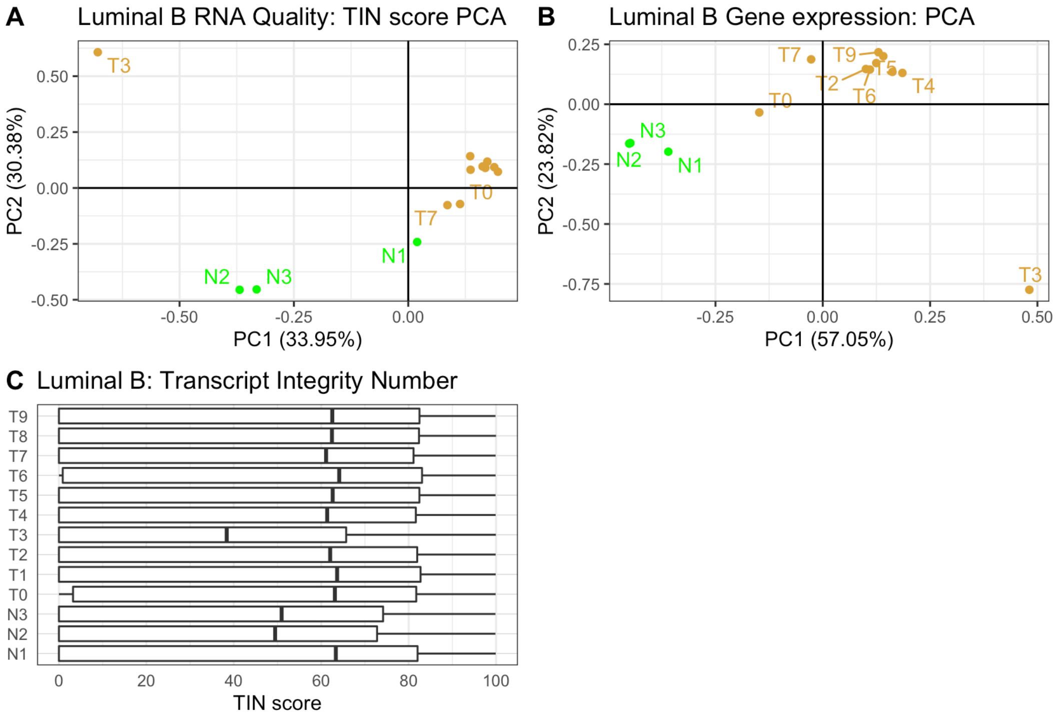

Figure S1: **A:** PCA of TIN score for 10 tumour and 3 normal samples for the single luminal B breast cancer patient. **B:** PCA of gene expression counts across tumour and normal tissue samples. **C:** Boxplot showcasing luminal B breast cancer patient TIN score for both tumour and normal tissue samples. In all cases, sample T3 is clearly identified as an outlier.

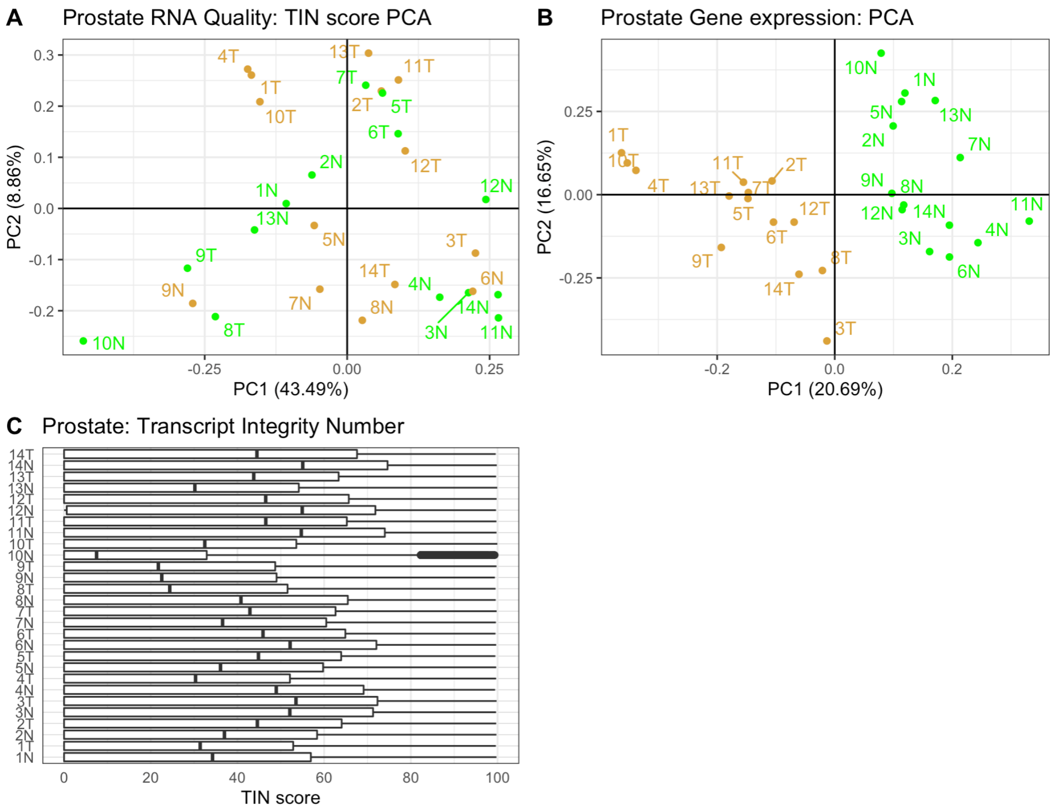

Figure S2: Prostate Dataset: A: PCA of TIN score for tumour and normal tissue samples across 14 patients. **B:** PCA of gene expression counts for tumour and normal tissue samples. **C:** Boxplot showcasing prostate cancer patient TIN scores for both tumour and normal tissue samples.

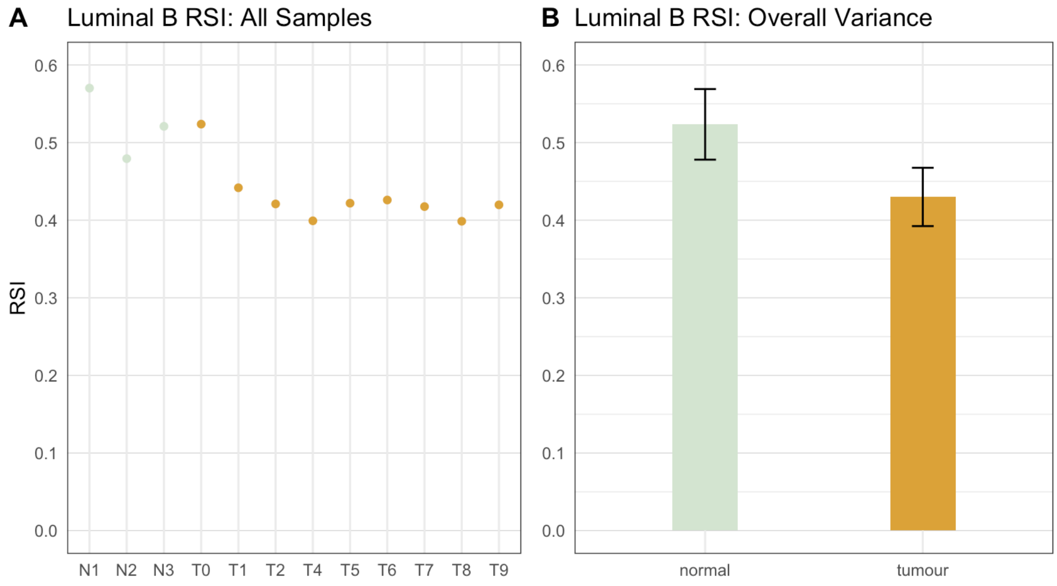

Figure S3: Luminal B Breast Cancer: A: Scatterplot showcasing RSI across tumour and adjacent normal tissue samples. **B:** Boxplot showcasing overall variance across tumour and normal tissue samples RSI.

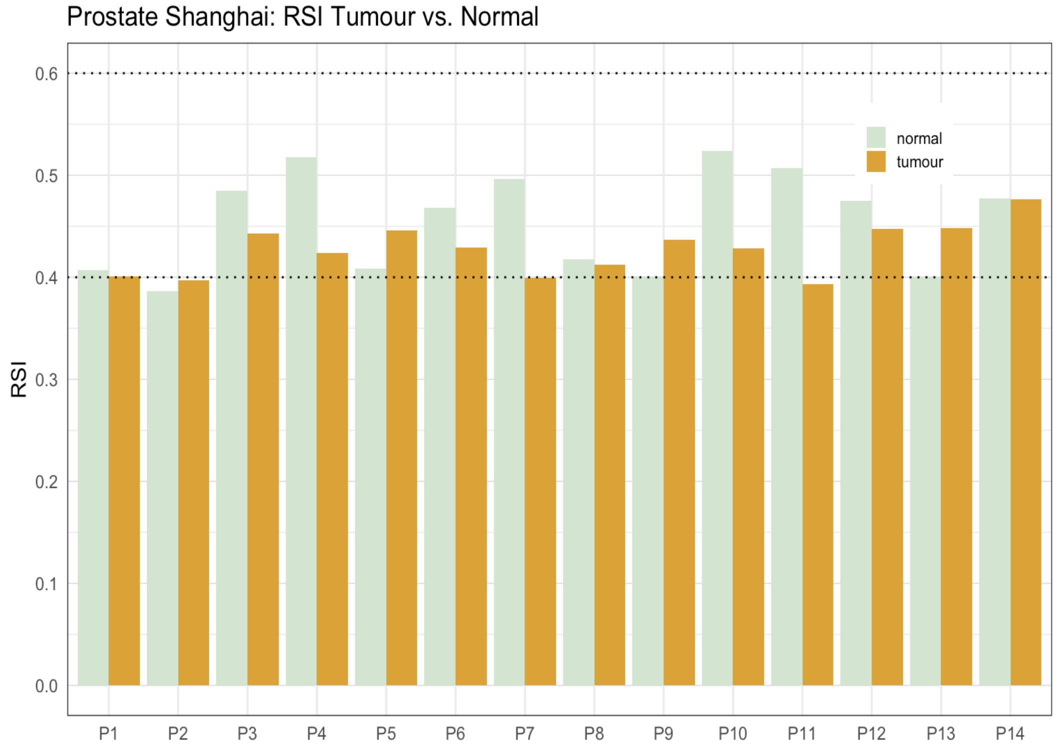

Figure S4: Prostate Cancer RSI for tumour and adjacent normal tissue in 14 normal-tumour matched samples. Dotted lines indicate the threshold RSI/SF2 (survival fraction at 2Gy) suitable for hypofractionation previously found to be within 0.4 – 0.6 [31]
